# Supplementary figures and images for: Transcranial magnetic stimulation alters multivoxel patterns in the absence of overall activity changes
Source: Hum Brain Mapp. 2021 May 15;42(12):3804–20. doi: 10.1002/hbm.25466 (PMC8288086; doi:10.1002/hbm.25466)

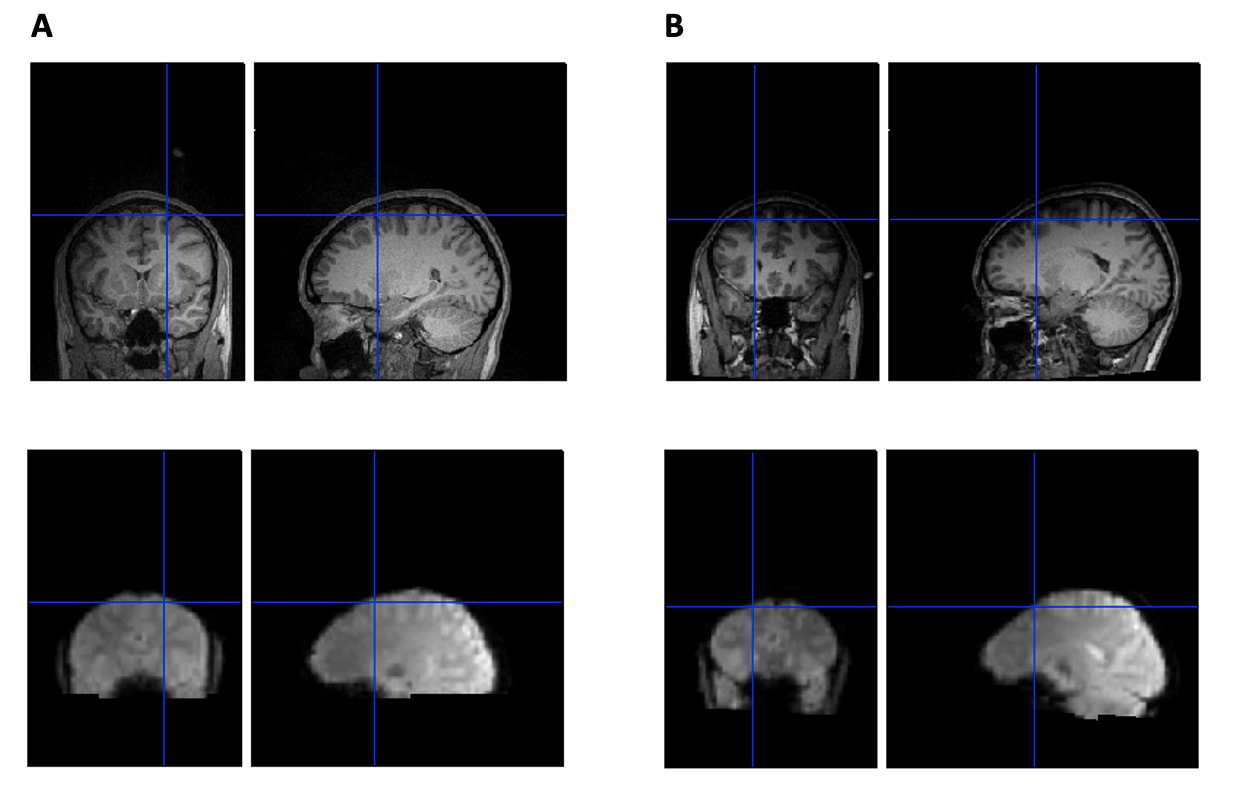

Supplement: Supplementary file 1 — Supplementary Figure 1 Quality of alignment of functional and anatomical images. The blue cross in all images shows the stimulation spot for a representative subject from (A) Experiment 1 and (B) Experiment 2. The top row shows the anatomical image of the subject, whereas the bottom row shows one volume from the functional scans. The quality of alignment was checked for all the subjects visually using SPM and no systematic deviation was found. The figure also shows that there was no visible dropout or distortion in the functional images at the site of stimulation that could have been caused by the presence of the TMS coil. [file HBM-42-3804-s003.png]

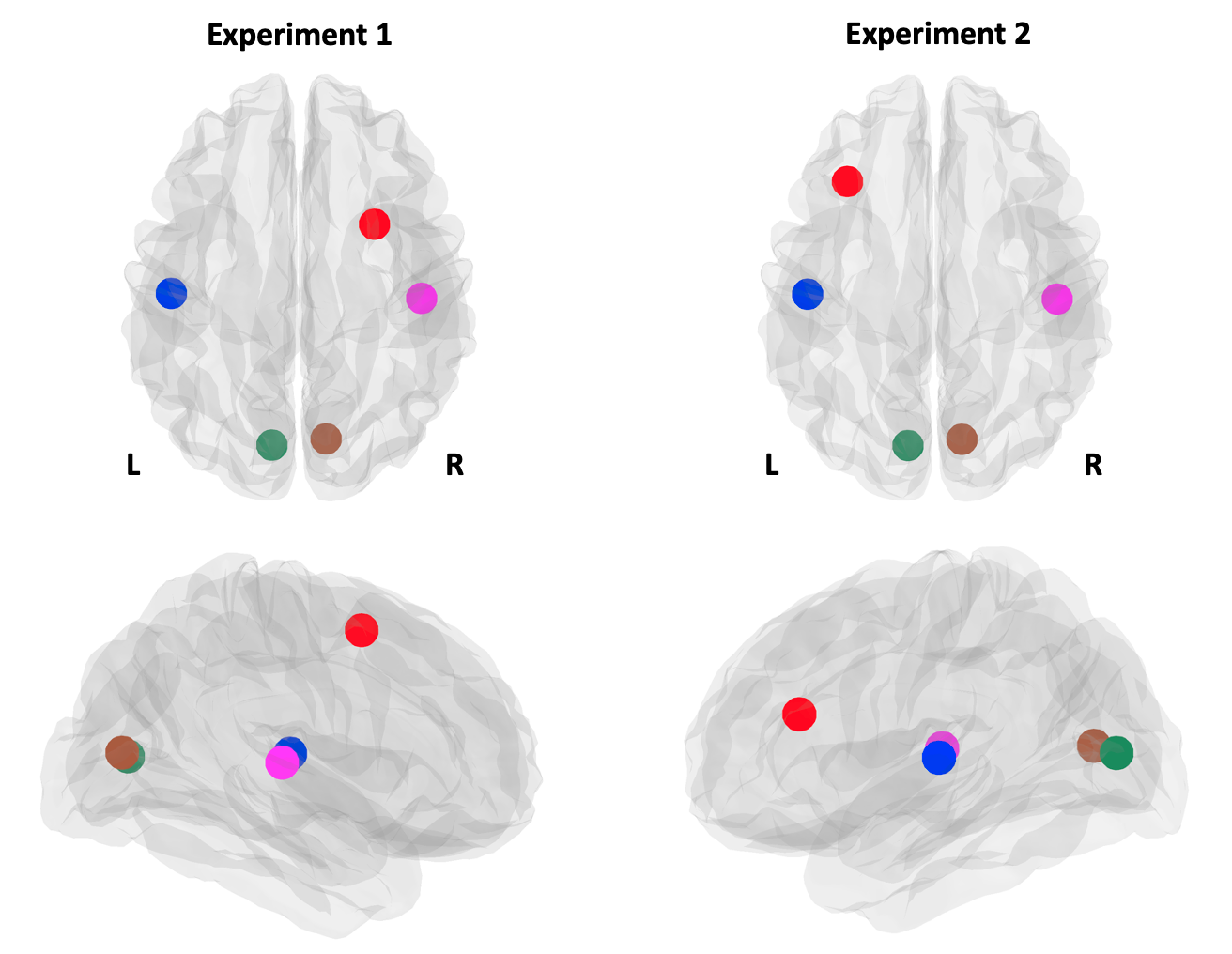

Supplement: Supplementary file 2 — Supplementary Figure 2 Visualization of ROI locations. The figure shows the centers of the spherical ROIs for subject 1 in Experiment 1 (left) and subject 1 in Experiment 2 (right). The green and brown dots correspond to the left and right visual cortex, respectively, whereas the blue and purple dots correspond to the left and right auditory cortex, respectively. Finally, the red dot shows the center of the 20‐mm ROI defined at the site of stimulation (note that the centers of the smaller ROIs defined at the site of stimulation would be slightly closer to the surface of the brain). [file HBM-42-3804-s002.png]

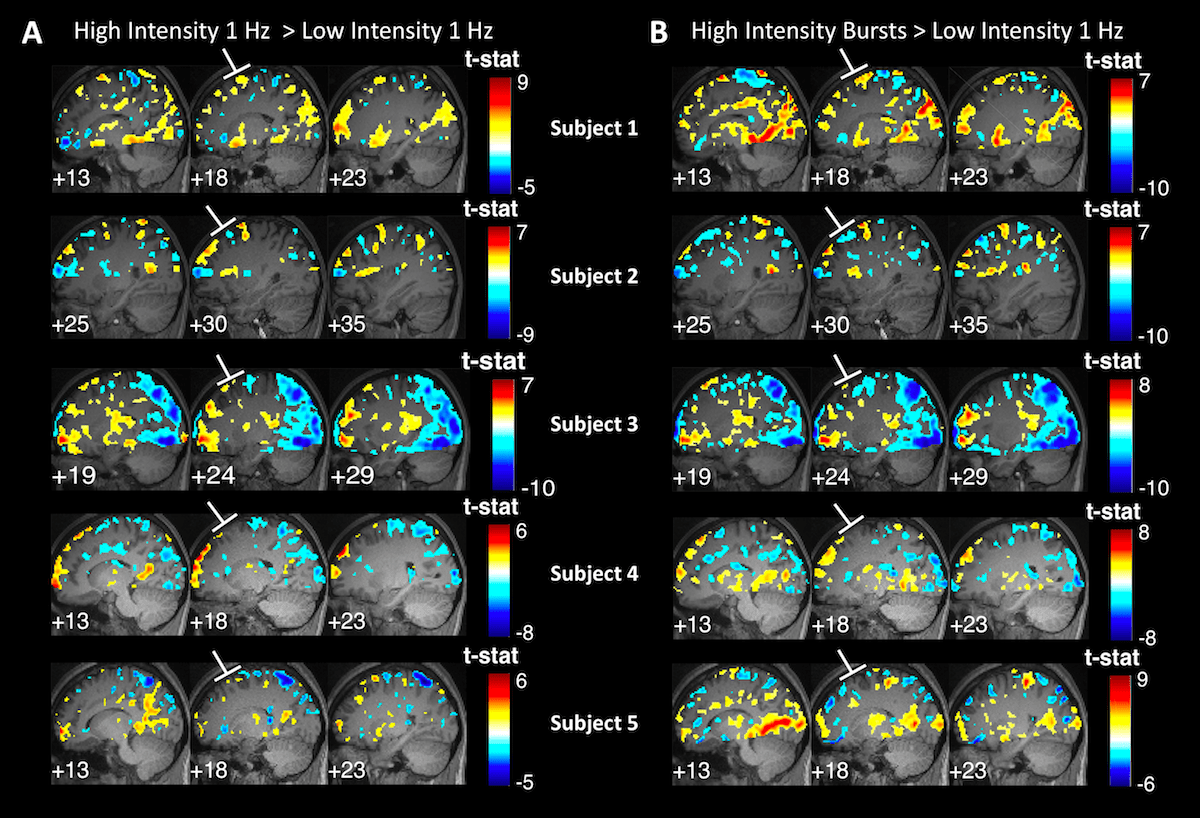

Supplement: Supplementary file 3 — Supplementary Figure 3 TMS to DLPFC produces no activation in the vicinity of the targeted area even at p < .05 uncorrected. We contrasted each of the two high‐intensity TMS conditions against the low‐intensity TMS condition, but, unlike in Figure 3, using an even more liberal threshold for significance (p < .05, uncorrected). (A) High intensity 1 Hz > Low intensity 1 Hz contrast. (B) High‐intensity bursts > Low intensity 1 Hz contrast. The activation maps show a lack of systematic activation in the vicinity of the targeted area and suggest the presence of large variability in remote areas across subjects. All figure details are as in Figure 3. [file HBM-42-3804-s005.png]

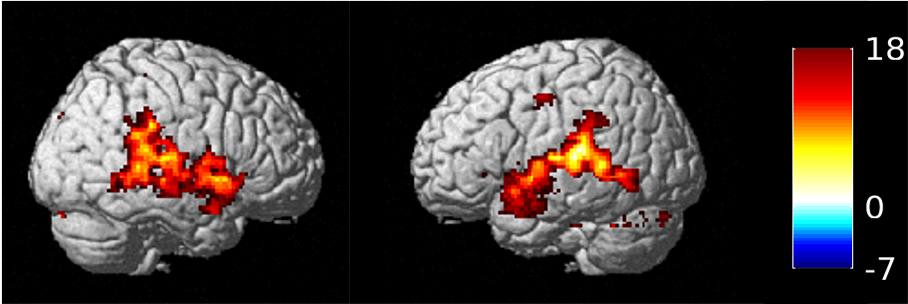

Supplement: Supplementary file 4 — Supplementary Figure 4 Univariate whole‐brain analysis in Experiment 2. A whole brain, second‐level analysis on the normalized data from all subjects using the contrast TMS > baseline revealed consistent TMS‐related activations in auditory cortices in Experiment 2. These activations were likely due to the click sound produced by TMS while stimulation. We note that similar analyses could not be run in Experiment 1 because we did not have whole‐brain coverage. For display purposes, the figure shows activations at p < 0.001, uncorrected. [file HBM-42-3804-s004.png]

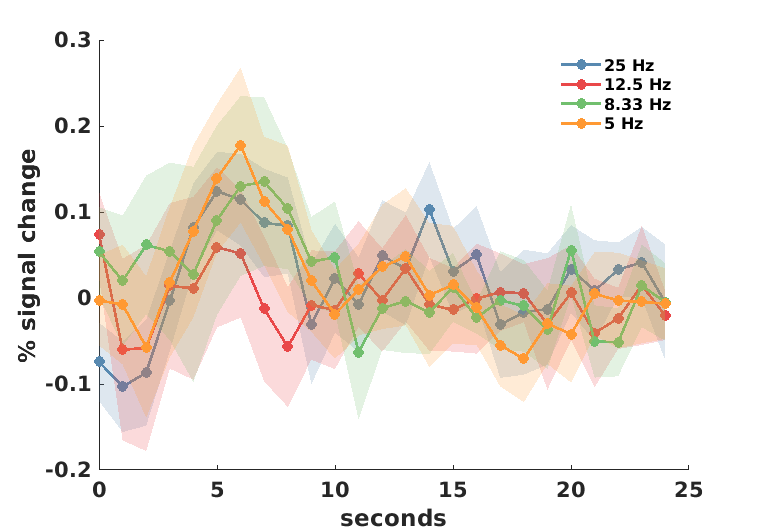

Supplement: Supplementary file 5 — Supplementary Figure 5 FIR analysis in Experiment 2. The figure plots the percentage of signal change as a function of time‐based on an FIR analysis in the 8‐mm spherical ROI defined at the site of stimulation. There was no significant difference between the overall time courses for the four conditions at any time point. Each data point shows the average percentage of signal change for all subjects. Zero is the time of stimulus onset and the shadowed areas depict s.e.m. [file HBM-42-3804-s006.png]

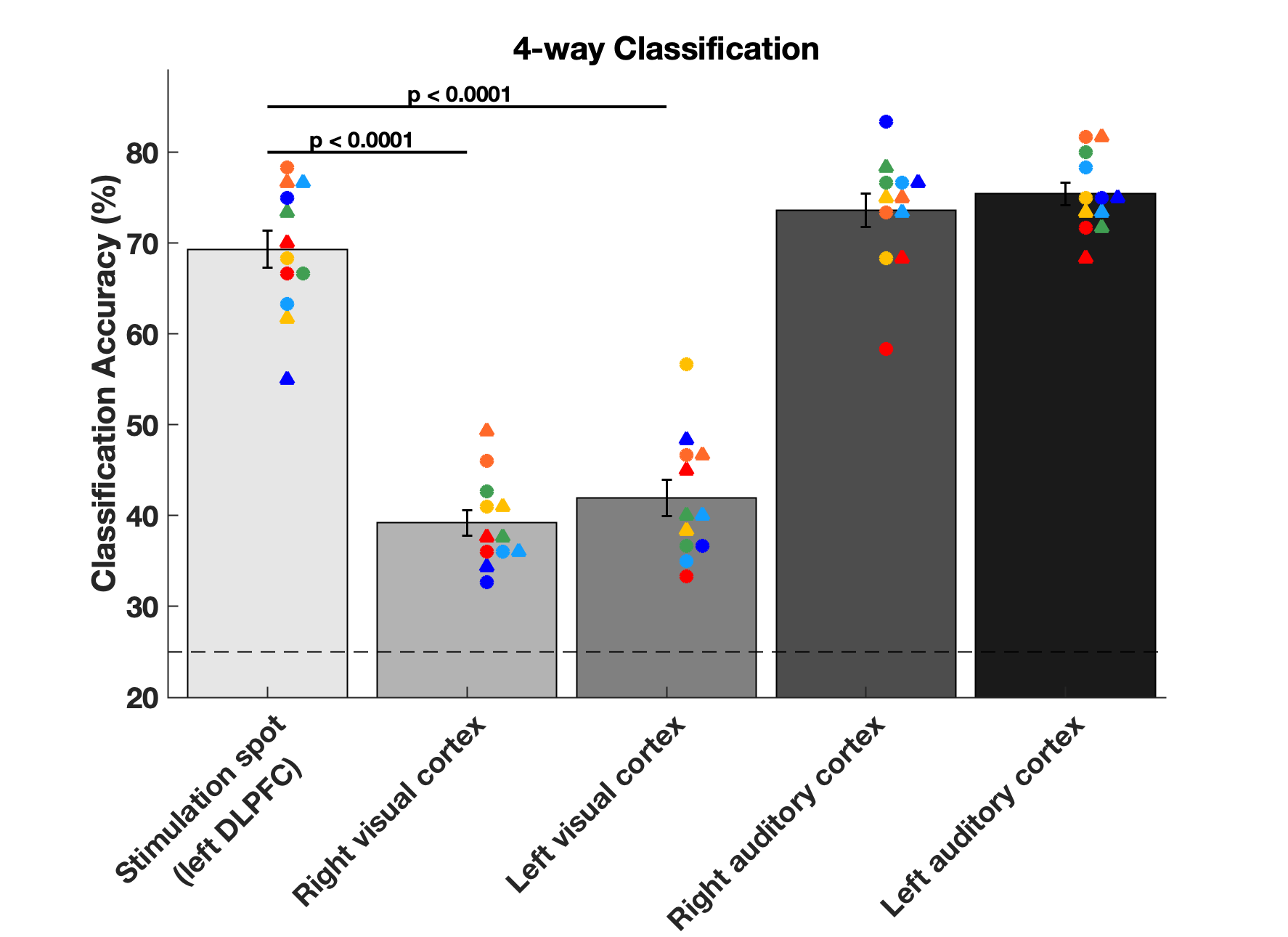

Supplement: Supplementary file 6 — Supplementary Figure 6 Decoding the TMS frequency using MVPA in different parts of the brain. We defined 20‐mm spherical ROIs in the left and right visual cortex, as well as left and right auditory cortex. The decoding performance at the stimulation spot was significantly greater than the decoding performance in the left and right visual cortex. In addition, decoding performance was very high in both the left and right auditory cortex presumably due to the clicking sounds produced by the TMS pulses. Error bars represent SEM, colors represent unique subjects with data from Day 2 plotted as a circle, and data from Day 3 plotted as a diamond. Chance performance is 25% (dashed line). [file HBM-42-3804-s001.png]
